# Dr. Alder, on Contagion and Fever

**Published:** 1807-11

**Authors:** Thomas Alder


### *To the Editors of the Medical and Physical Journal.*

GENTLEMEN,

As the following doctrines, particularly so far as they relate to Quarantine, will, if acted on, lead to immense alterations, I request not that you or your numerous readers should pay me the compliment to adopt them without anxious thought, but that you will give them a dispassionate and careful examination: in doing this, some, perhaps, may say I shew a distrust of what I write? But in return, I can assure them, I feel no other distrust than what a man should feel when he relies altogether on his own judgment; and that though I solicit to be consi-

 dered dered as an advocate rather than as a judge, I wish the train of facts and arguments which I have to adduce, may meet with candour, as I have never to my knowledge misstated or misrepresented the smallest circumstance, or given to an argument more force than it was entitled to.

In the last letter which I had the honour to address to you, (published in your Journal for June) I took a glance at the declension of our trade, the failure of our revenue, and a national bankruptcy, as effects which might result from our Quarantine doctrines: and promised to shew such doctrines were totally erroneous; I mean in this and my succeeding letters to go over some of the same grounds again more in detail; and to endeavour to fulfil my promise.

In my last, I noticed as an important circumstance, that fevers have prevailed over almost all the known world: they have *often* done this,—I think according to Mr. Noah Webster's learned and valuable history of Epidemics, they have done so once in fifteen or twenty years, for some thousand years: and they have sometimes continued to prevail a long time together; but it is rather difficult in most cases to say exactly, how far a general epidemic does extend. If, however, it extend to Syria, Egypt, the other shores of the Mediterranean, our settlements in Africa, the West Indies, the United States of America, and the East Indies, it will extend to most of the important places which we trade to; and those are the places which it has most commonly attacked.

Now, according to this statement, (and it must be evident it is true) I do not see how we can expect our trade to be carried on to these places to that extent *which has now become necessary to our existence as a nation*, for very many years uninterruptedly, according to the quarantine doctrines; but happily for the country, these doctrines are opposed by experience; and consequently may be somewhat disregarded.

It is inconsistent in medical men, who maintain that common fever is contagious, to assert the endemics of Syria, of Egypt, of our settlements in Africa, of the West Indies, the United States of America, of Bombay, and the other parts of the East Indies, and of the Island of Java, are not contagious, because, the common fever seldom prevails rarely so *extensively* as they do, and is not comparably so dreadful? In fact, the advocates for contagion find a great reluctance in admitting any of these to be non-contagious; but a long intercourse with these places during their their endemics, joined with the opinion of the natives, has induced a hope in many, that the common fevers of most of these places are *not contagious*; and a communication is carried on with them quite contrary to that caution which the strict advocates for quarantine require; this is one reason why we as a nation have suffered so little already.

But our security ought not and cannot depend upon practices which are the result of any thing but a clear and definite knowledge; the medical men who advise government may say even now, such practices are wrong; I know there are many of the greatest eminence, who stand aghast at the idea of the common fever of Bengal, of the West Indies, and of America, not being deemed contagious, and who would not let a ship come near our shores from any place where a fever prevailed at its departure! Tho' but few places which we most trade to, are ever exempt from some fever for any length of time, what would most medical men then have, if *epidemic* fevers raged at those places. For "*a widely spreading epidemic*" with them signifies the same as "*a highly contagious disorder.*" I will not say what such men might wish to have done; they, perhaps, might wish to burn all the ships which ever had been at such places?—But, if they only got Quarantine strictly enforced, they might do an injury which our trade and revenue could *never* recover; and more particularly if other nations should disregard Quarantines. But indeed such gentlemen, to be *consistent*, should burn our ships and their cargoes; for I cannot admit that Quarantine could cleanse them from contagion! Under our present theoretic ideas then, we cannot be sure that a *practical* consciousness of safety in trading to most places under certain circumstances will be sufficient to warrant us in the authority freely to continue to trade to such places under such circumstances; and we may fearfully apprehend circumstances will soon and often arise, which will render it almost, if not altogether, impossible to trade to such places for a great length of time.

If what I take the liberty to offer, is so new,* and on subjects so highly interesting, I think you will agree with me, I shall do best to write but little at a time, in order to give the utmost opportunity to your Correspondents,

* Dr. A. will see in our 102 No. page 111, that his ideas have been familiar in America for a considerable time. Ed.

dents, to consider and oppose it. It is evident, I anticipate contradiction; but I hope envy will have no share in it, as any popularity or distinction which it is *possible* for me to gain, will have cost more than any body would choose to give for it, and I may be charged with causing evils, towards the induction of which my intention will be totally guiltless.

I am, &c

THOMAS ALDER, M. D.

*October* 2, 1807.

